# Beneficial effects of choir singing on cognition and well-being of older adults: Evidence from a cross-sectional study

**DOI:** 10.1371/journal.pone.0245666

**Published:** 2021-02-03

**Authors:** Emmi Pentikäinen, Anni Pitkäniemi, Sini-Tuuli Siponkoski, Maarit Jansson, Jukka Louhivuori, Julene K. Johnson, Teemu Paajanen, Teppo Särkämö

**Affiliations:** 1 Cognitive Brain Research Unit, Department of Psychology and Logopedics, Faculty of Medicine, University of Helsinki, Helsinki, Finland; 2 Department of Music, Art and Culture Studies, University of Jyväskylä, Jyväskylä, Finland; 3 Institute for Health & Aging, University of California San Francisco, San Francisco, California, United States of America; 4 Finnish Institute of Occupational Health, Helsinki, Finland; Medical University of Innsbruck, AUSTRIA

## Abstract

**Background and objectives:**

Choir singing has been associated with better mood and quality of life (QOL) in healthy older adults, but little is known about its potential cognitive benefits in aging. In this study, our aim was to compare the subjective (self-reported) and objective (test-based) cognitive functioning of senior choir singers and matched control subjects, coupled with assessment of mood, QOL, and social functioning.

**Research design and methods:**

We performed a cross-sectional questionnaire study in 162 healthy older (age ≥ 60 years) adults (106 choir singers, 56 controls), including measures of cognition, mood, social engagement, QOL, and role of music in daily life. The choir singers were divided to low (1–10 years, N = 58) and high (>10 years, N = 48) activity groups based on years of choir singing experience throughout their life span. A subcohort of 74 participants (39 choir singers, 35 controls) were assessed also with a neuropsychological testing battery.

**Results:**

In the neuropsychological testing, choir singers performed better than controls on the verbal flexibility domain of executive function, but not on other cognitive domains. In questionnaires, high activity choir singers showed better social integration than controls and low activity choir singers. In contrast, low activity choir singers had better general health than controls and high activity choir singers.

**Discussion and implications:**

In healthy older adults, regular choir singing is associated with better verbal flexibility. Long-standing choir activity is linked to better social engagement and more recently commenced choir activity to better general health.

## Introduction

As the population ages and the proportion of older adults grows, it has become increasingly important to find new ways to support and improve their quality of life and well-being. Not only age-related neurodegenerative diseases, but also normal aging induces many changes in brain function and cognition as well as in social and physical conditions of life that can significantly affect individual’s well-being. Cognitively, healthy aging is known to affect primarily abilities that require flexible recruitment and modification of task-appropriate skills, while general knowledge acquired earlier in life is preserved [1]. Processing speed and executive functions, such as inhibitory control and selective attention, seem to be vulnerable to age-related changes in the brain [2]. These changes in cognition can be linked to atrophy in specific brain areas, including the prefrontal cortex, occurring with normal aging [3]. Cognitive decline is often linked also to emotional deficits, and, for example, depression is a common reactive symptom in the early stages of dementia [4]. On the other hand, depression has been identified as an important risk factor for cognitive decline and dementia [5]. Socially, aging is also associated with reduced social networks and increased loneliness, which can seriously impair both cognitive and emotional well-being as well as physical health [6, 7]. Overall, as cognitive, emotional, and social well-being are so closely intertwined, it is of great importance to find new versatile ways to enhance the general well-being of older adults.

Although age-related changes occur, the brain remains plastic throughout life and ageing is not associated only with negative neural changes: the aging brain can adapt structurally and functionally to compensate for lost volume and functioning of specific areas, for example by recruiting frontal areas more extensively and reducing bilateral asymmetry in carrying out functions [8]. Evidence from intervention studies suggests that cognitive training can have positive effects on cognition and brain structure and function in older adults [8, 9]. Similarly, research on cognitive reserve indicates that there are a number of protective lifestyle-related factors, such as education and cognitive, social, and physical activities, that can help preserve cognitive functioning even when neurodegenerative changes are already apparent [5]. There is now growing interest towards everyday activities that can help preserve cognitive function in old age. Physical exercise is perhaps the most studied of these, and there is mounting evidence that it can decrease the risk of cognitive decline and dementia as well as slow down age-related neural changes [10]. Recently, attention has turned also to the role of cultural activities and arts, especially those involving music, as effective tools to improve health and well-being, in terms of prevention and management of illnesses [11] and in the context of aging [12].

### Musical activities and healthy aging

Converging findings from psychological, clinical, and neuroimaging research suggest that music is an important source of enjoyment, learning, and well-being as well as a versatile stimulus for the brain, also during aging [13]. Music is commonly used to regulate mood and arousal, communicate and interact with others, and enrich everyday life. This can be observed also in times of crisis; for example, during the COVID-19 pandemic, people quarantined to their homes turned to playing music and singing together from their open windows and balconies to help cope with emotional distress and social isolation. In the brain, music engages multiple cognitive, motor, emotional, and social processes mediated by a wide-scale, largely bilateral network of cortical and subcortical brain regions [14, 15]. Musical training has been found to enhance cognitive performance, with transfer effects on executive functions, attention, and memory [16, 17], and induce structural and functional neuroplasticity changes [18], especially in temporal, frontal, parietal, and cerebellar regions associated with higher-level auditory-cognitive functions [19]. Notably, some evidence suggests that prefrontal cortical regions, which are most vulnerable to age-related atrophy, are more preserved in older musicians than in older control subjects [20] and that music making has an age-decelerating effect on brain structure [21]. Lifetime instrumental musical activities and training to play a musical instrument at old age have been linked to better cognitive flexibility, processing speed, working memory, and verbal and non-verbal memory [22–25].

### Choir singing and healthy aging

While the neurocognitive impact of instrumental musical training has been extensively studied, little is known about the potential effects of singing on neuroplasticity and cognitive functioning during aging. For the brain, singing is a highly versatile and multi-domain process, requiring the complex interplay of auditory, vocal-motor, linguistic, cognitive, and emotional processes. Neuroimaging studies suggest that singing entails continuous interaction between two cortical systems, the parietal-frontal (dorsal) vocal production pathway and the temporal-frontal (ventral) auditory perception pathway, which work together as a loop to enable fine vocal motor control based on somatosensory and auditory feedback [26]. In addition to these core systems, also other prefrontal, limbic, and cerebellar areas linked to attention, working memory, rhythm, and emotion are engaged during singing perception and production [27–29].

Among different musical activities, choir singing is the most popular and widespread hobby, also among seniors. In Europe, there are 37 million choir singers, and participation in senior choirs is growing rapidly [30]. The coupling of singing-related brain processes (vocal-motor, auditory, linguistic, cognitive, emotional) with the social interaction (singing together in a group) and goal-directed learning (learning to sing and perform polyphonic song arrangements) elements makes choir singing a particularly promising activity for promoting cognitive reserve and psychological and social well-being in aging. Previous research on group singing has shown that it can improve mental health and emotional and social well-being in adults who have a mental health condition [31]. Physiologically, singing has a positive impact on cardiorespiratory functions [32, 33], and the emotional gains of singing are linked to the secretion of endocannabinoids, immunoglobulins, and cortisol [34, 35]. In older adults, regular participation in community-level choirs can reduce anxiety, depression, and loneliness; improve self-evaluated quality of life (QOL), physical health, and interest in life; and increase general activity [36–39].

### Potential cognitive benefits of choir singing in older adults

Evidence for the potential cognitive benefits of choir singing in healthy older adults is scarce, thus far limited to a single study. In a pilot study of healthy older adults (N = 49), Fu et al. [33] reported improved performance in verbal fluency and memory tests after a 12-week group singing program, but lack of a control group limits the conclusions that can be drawn from this result. No longitudinal study has yet been conducted to explore the long-term effects of choir singing in the elderly. Here, we report baseline results of an ongoing longitudinal study in Helsinki where a cohort of elderly choir singers and non-singer control subjects are followed over a three-year period using questionnaires, neuropsychological tests, and electroencephalography (EEG) measurements. In this cross-sectional comparison of active choir singers and demographically matched controls (total N = 162), our hypotheses were that choir singers show better cognitive performance, especially in tasks measuring executive functions, as well as better self-reported mood, social well-being and QOL compared to the controls. Moreover, we sought to explore whether the length of the singing experience affected the potential benefits of choir singing.

## Design and methods

### Participants and study design

The study was approved by the Ethical Review Board in the Humanities and Social and Behavioural Science of the University of Helsinki. All participants gave written informed consent. The participants were 162 older adults recruited from the Adult Education Centers of the Cities of Helsinki, Espoo and Vantaa and from different senior citizens’ associations and independent choirs in the Helsinki area. Participants were recruited through presentations, flyers, and e-mail advertisements. The cross-sectional study reported here is from the baseline data of an ongoing longitudinal cohort study of the effects of senior choir singing on neurocognitive ageing. The present study comprises two parts: a main cohort study with questionnaire data from the full participant sample (N = 162) and a subcohort study with neuropsychological test data from a subsample of the participants (N = 74). The inclusion criteria in the main cohort study were (i) age ≥ 60 years, (ii) Finnish-speaking, and (iii) absence of neurological (e.g., dementia, stroke) or psychiatric (e.g., schizophrenia, bipolar disorder) disorders. In the subcohort study, additional inclusion criteria were (iv) absence of medication affecting CNS function, (v) absence of significant hearing loss, and (vi) absence of severe sleep disorder (e.g., insomnia, sleep apnea).

Of the 162 participants, 106 were choir singers (persons who currently sing in a choir and who had been singing for at least one year) and 56 were control subjects (persons who do not sing in a choir currently and have not participated in choir singing during the last 10 years). Choir singing was defined as regular participation in a choir, which (i) is led by a professional choir conductor, (ii) trains together at least once a week, and (iii) performs regularly (at least twice a year). For the main cohort study, the choir singers were divided to those who had started choir singing earlier in life and had sung in a choir for more than 10 years (referred to hereafter as “high activity choir singers”) and those who had started choir singing later in life and had sung in a choir for 10 years or less (hereafter “low activity choir singers”). A similar classification was used previously by Hanna-Pladdy and MacKay [24] in their study of older adult musicians. This resulted in three relatively balanced groups for the main cohort study: (1) high activity choir singers (N = 48), (2) low activity choir singers (N = 58), and (3) control subjects (N = 56). In the subcohort study, the participants were choir singers (N = 39) and control subjects (N = 35).

### Neuropsychological testing

Neuropsychological testing was conducted for a randomly selected subsample (N = 74) of the main cohort. The testing (duration 1.5 h) was performed by a trained psychologist in a quiet testing room. The testing battery (see Table 1 for details) covered six cognitive domains: general cognition, executive functions, processing speed, working memory, episodic memory, and verbal skills. General cognition was evaluated with the Montreal Cognitive Assessment (MoCA) [40]. Executive functions were further divided to three subdomains: (i) verbal flexibility assessed with the Phonemic fluency test [41]; (ii) shifting assessed with a computerized (tablet) modification of the Trail Making Test, which is included in the Flexible Attention Test (FAT) developed at the Finnish Institute of Occupational Health; and (iii) inhibition assessed with a tablet version of the Simon task [42]. Processing speed was evaluated with the Symbol search and Coding subtests of the Wechsler Adult Intelligence Scale IV (WAIS-IV) [43]. Working memory was assessed with the Digit span subtest of WAIS-IV and a tablet version of the Corsi Block-tapping test [44]. The Arithmetic subtest of WAIS-IV was used as a separate measure of problem solving. Episodic memory was evaluated using the Logical memory and Word lists subtests of the Wechsler Memory Scale III (WMS-III) [45]. Verbal skills were evaluated with the Vocabulary subtest from WAIS-IV and with the Semantic fluency task [41]. For each domain, sum scores of the individual tests were used in the analyses.

**Table 1:** Description of outcome measures.

Type	Domain	Measure	Description	Variables
Q	Cognitive function	CFQ	25 items measuring cognitive failures in different everyday situations involving perception, attention, memory and motor functions	total score (sum)
		PRMQ	16 items measuring prospective and retrospective memory	total score (sum)
Q	Depression	CES-D	20 items measuring depressive symptoms	total score (sum)
Q	Social well-being	SPS	24 items measuring level of support from social relationships	total score (sum)
			6 scales: Attachment, Social Integration, Reassurance of Worth, Reliable Alliance, Guidance, and Opportunity of Nurturance	6 scale scores (sum)
Q	QOL	WHOQOL-Bref	26 items measuring different aspects of quality of life	
			4 scales: Physical, Psychological, Social, and Environmental QOL	4 scale scores (sum)
			2 separate items: Overall QOL and General Health	2 item scores
Q	Role of music	MusEQ	35 items measuring the use of music and its role in everyday life	total score (weighted)
T	General cognition	MoCA	6 short tasks measuring visuospatial functions, verbal abilities, memory, attention and orientation	total score (sum)
T	EF: Verbal Flexibility	Phonemic fluency	List verbally as many words as possible during 60 seconds starting with the letter S	score
T	EF: Shifting	FAT	Trail Making Test: Connect numbers (Part A) and numbers and letters (Part B) as fast as possible	time difference (B-A)
T	EF: Inhibition	Simon task	Respond to red/blue square appearing on the left/right side of screen by pressing a button [congruent (CON) and incongruent (INC) trials]	time difference (INC-CON)
T	Processing speed	WAIS-IV	Visual Search: Copy symbols corresponding to numbers (2 min)	sum of raw scores
			Coding: Search rows of symbols for target symbols (2 min)	
T	Working memory	WAIS-IV	Digit span. Recall lists of numbers in different order	sum of raw scores
		FAT	Visual span: Recall visuospatial patterns	
T	Problem solving	WAIS-IV	Arithmetic: Solve verbally presented arithmetic tasks	raw score
T	EM: Immediate	WMS-III	Logical memory I: Recall a story immediately after hearing it	sum of raw scores
			Word lists I: Recall a list of words immediately after hearing it	
T	EM: Delayed	WMS-III	Logical memory II: Recall a story after 30 min delay	sum of raw scores
			Word lists II: Recall a list of words after 30 min delay	
T	Verbal skills	WAIS-IV	Vocabulary: Explain the meaning of words	sum of raw scores
		Semantic fluency	List verbally as many animals as possible during 60 seconds	

Abbreviations: CES-D = Center for Epidemiologic Studies Depression scale, CFQ = Cognitive Failures Questionnaire, EF = executive function, EM = episodic memory, FAT = Flexible Attention Test, MoCA = Montreal Cognitive Assessment, MusEQ = Music Engagement Questionnaire, PRMQ = Prospective and Retrospective Memory Questionnaire, Q = questionnaire, SPS = Social Provisions Scale, T = neuropsychological test, WAIS-IV = Wechsler Adult Intelligence Scale IV, WHOQOL-Bref = Quality of Life Questionnaire of the World Health Organizations, WMS-III = Wechsler Memory Scale III.

### Questionnaires

In the main cohort study (N = 162), six self-report questionnaires (see Table 1 for details) were used to measure cognitive functioning, depression, social well-being, QOL, and role of music in daily life. Cognitive functioning was assessed with the Cognitive Failures Questionnaire (CFQ) [46] and the Prospective and Retrospective Memory Questionnaire (PRMQ) [47]. Depression was evaluated using the Center for Epidemiologic Studies Depression scale (CES-D) [48]. Social well-being was assessed with the Social Provisions Scale (SPS) [49]. QOL was assessed with the Quality of Life Questionnaire of the World Health Organizations (WHOQOL-Bref) [50]. Role of music in daily life was evaluated with the Music Engagement Questionnaire (MusEQ) [51].

As a background variable, level of cognitive and physical activity was assessed with six items from the Lifetime of Experiences Questionnaire (LEQ) [52], three concerning cognitive activity (non-musical arts, reading, language learning) and three concerning physical activity (mild, moderate and vigorous exercise) during old adulthood (from age 60 years). Also the frequency of group singing during young adulthood, middle age, and old adulthood was measured with an additional item of the LEQ questionnaire.

### Statistical analyses

Statistical analyses were performed with SPSS (IBM SPSS Statistics 25). Group differences in demographic background variables were analyzed with independent-samples t tests, one-way ANOVAs, and chi-square tests. Group differences in the outcome measures (questionnaires and neuropsychological tests) were analyzed with univariate ANCOVAs where those background variables, which showed group differences, were included as covariates. Post hoc tests were performed using the least significant difference (LSD) test.

## Results

### Demographic characteristics of the participants

The demographic characteristics of the participants are shown in Table 2. In the main cohort study, there were significant or marginally significant differences between the three groups in age [F(2,159) = 5.76, p = 0.004], gender [χ^2^ (2) = 6.74, p = 0.034], and living situation [χ^2^ (2) = 5.76, p = 0.056]. The high activity choir singers were on average older than the low activity choir singers (p = 0.001) and the controls (p = 0.019). The low activity choir singers had smaller proportion of women (p = 0.012) and persons living alone (p = 0.018) than the controls. In the subcohort study, marginal differences were seen in education level [t(62.67) = 1.82, p = 0.074] and gender [χ^2^ (1) = 2.53, p = 0.111], with slightly higher education level and proportion of women in the control group. In the ANCOVAs of outcome measures, age, gender, and living alone were included as covariates in the main cohort study and education level and gender as covariates in the subcohort study.

**Table 2:** Demographic characteristics of the participants.

	Questionnaire study				Neuropsychological study		
	High activity choir singers (N = 48)	Low activity choir singers (N = 58)	Control subjects (N = 35)	p value	Choir singers (N = 39)	Control subjects (N = 35)	p value
Age (years)	72.8 (5.7)	69.2 (4.5)	70.2 (6.5)	0.004 (F)	70.9 (5.9)	69.7 (6.6)	0.396 (t)
Gender (women / men)	38 / 10	38 / 20	48 / 8	0.034 (χ^2^)	26 / 13	29 / 6	0.111 (χ^2^)
Education level^a^	4.3 (2.2)	4.2 (1.9)	4.7 (2.1)	0.375 (F)	3.8 (1.7)	4.7 (2.3)	0.074 (t)
Living situation (alone / together)	20 / 27	16 / 41	27 / 27	0.056 (χ^2^)	15 / 24	17 / 18	0.381 (χ^2^)
Cognitive activity (old adulthood)^b^	13.2 (3.0)	12.7 (2.6)	12.4 (3.7)	0.446 (F)	12.5 (2.4)	12.6 (3.9)	0.857 (t)
Physical activity (old adulthood)^c^	9.1 (2.4)	9.7 (2.8)	9.3 (3.1)	0.558 (F)	9.7 (2.4)	9.8 (3.4)	0.895 (t)
Group singing (old adulthood)^d^	3.9 (0.3)	4.0 (0.4)	0.9 (1.4)	<0.001	4.0 (0.5)	0.6 (1.1)	<0.001
Group singing (middle age)^d^	3.6 (1.0)	1.6 (1.5)	0.8 (1.4)	<0.001	2.6 (1.6)	0.9 (1.5)	<0.001
Group singing (young adulthood)^d^	3.3 (1.3)	1.9 (1.5)	1.0 (1.4)	<0.001	2.6 (1.6)	0.8 (1.3)	<0.001
Group singing years total	27.3(14.2)	6.3 (2.8)		<0.001	15.8(14.3)		

Data are mean (SD) unless otherwise reported. Abbreviations: F = one-way ANOVA, χ^2^ = chi-square test, t = independent-samples t test.

^a^Education level according to the Unesco International Standard Classification of Education: range 1 (primary education) to 8 (doctoral level).

^b^Cognitive activity level during older adulthood based on the Lifetime of Experiences Questionnaire (LEQ) scores.

^c^Physical activity level during older adulthood based on the Lifetime of Experiences Questionnaire (LEQ) scores.

^d^Self-rating of group singing activity on a 6-point Likert scale ranging from 0 (never) to 5 (daily).

The choir singers and control subjects were well-matched for the level of non-musical cognitive and physical activities. In the main cohort study, the three groups differed highly significantly in the frequency of group singing at old age [age ≥ 60 years; F(2,154) = 209.34, p<0.001], middle age [age 30–59 years; F(2,154) = 56.84, p<0.001], and young age [age 13–29 years; F(2,154) = 32.81, p<0.001]. Post hoc tests showed that both the high activity and low activity choir singing groups reported more frequent group singing than the control group at each life era (p<0.005 in all) but did not differ from each other in group singing at old age (p = 0.948). The high activity choir singers had more frequent group singing than the low activity choir singers at middle (p<0.001) and young (p<0.001) ages, confirming that the low activity choir singers had indeed started their choir hobby mostly after the age of 60 years. In the subcohort study, the choir singers had clearly more frequent group singing activity than the control subjects at each life era (p<0.001 in all).

### Neuropsychological test results

**Fig 1:**
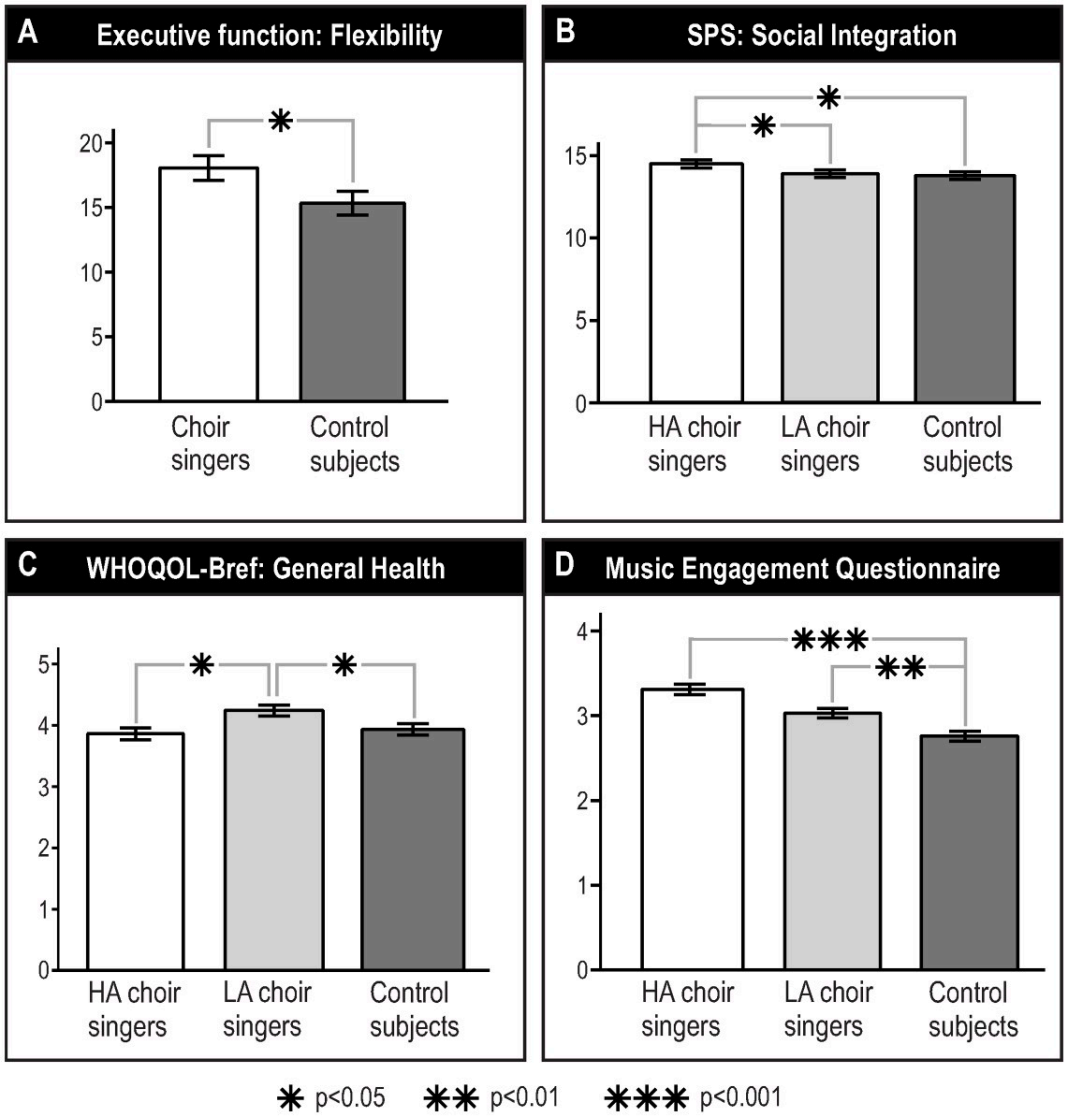
Significant group differences between choir singers and control subjects in (A) verbal flexibility, (B) social integration, (C) general health, and (D) music engagement. Bars show data as mean (SEM), with the significance level of the pair-wise group differences indicated with asterisks. Abbreviations: HA = high activity, LA = low activity.

The neuropsychological test scores of the choir singer and control groups are shown in Table 3. As illustrated in Fig 1A, the choir singers were significantly better than the controls on the verbal flexibility subdomain of executive functions [F(1,64) = 4.09, p = 0.047]. There were no significant group differences in the other cognitive domains. For those tests which are standardized and have adequate Finnish normative data, and for which a cut-off value for impaired performance can be established (visual search, coding, digit span, arithmetic, word lists, vocabulary), there were no relevant differences between the choir singer and control groups on the proportion of participants scoring in the impaired range (see S1 Table, online only).

**Table 3:** Group differences in neuropsychological tests.

Questionnaire	Choir singers (N = 39)	Control subjects (N = 35)	p value^a^
General cognition	25.9 (2.7)	25.7 (2.9)	0.275
Executive function: Verbal Flexibility	17.3 (5.7)	16.0 (5.2)	**0.047**
Executive function: Shifting	37.3 (26.6)	36.9 (23.8)	0.848
Executive function: Inhibition	101.2 (44.1)	98.0 (47.1)	0.912
Processing speed	70.6 (16.3)	75.3 (16.1)	0.637
Working memory	34.03 (4.8)	33.51 (5.6)	0.469
Arithmetic	13.24 (3.3)	13.37 (3.3)	0.619
Episodic memory: Immediate recall	41.0 (9.7)	42.3 (8.5)	0.961
Episodic memory: Delayed recall	16.3 (6.7)	16.4 (6.2)	0.646
Verbal skills	60.1 (11.8)	59.5 (12.9)	0.151

Data shown as mean (SD).

^a^The p value is the Group effect from univariate ANCOVA with education level and female gender as covariates. Significant effects shown in bold font.

### Questionnaire results

The questionnaire scores of two choir singer groups and the control group are shown in Table 4. Significant group differences were observed in three questionnaires: the Social Integration scale of SPS [F(2,151) = 3.15, p = 0.046; Fig 1B], the General Health item of WHOQOL-Bref [F(2,151) = 3.61, p = 0.029; Fig 1C], and the MusEQ total score [F(2,150) = 8.53, p<0.001; Fig 1D]. There were no other significant effects. Post hoc tests indicated a different pattern of pair-wise group differences in the three questionnaires. In SPS Social Integration, the scores were higher in the high activity choir singers than in the low activity choir singers (p = 0.037) and control subjects (p = 0.023) whereas the low activity choir singers and control subjects did not differ (p = 0.850). In contrast, the WHOQOL-Bref General Health scores were higher in the low activity choir singers than in the high activity choir singers (p = 0.014) and control subjects (p = 0.037) but did not differ between the high activity choir singers and control subjects (p = 0.637). Finally, the MusEQ scores were higher in both the high activity and low activity choir singers compared to the control subjects (p<0.001 and p = 0.009, respectively), but did not differ between the two choir singer groups (p = 0.136).

**Table 4:** Group differences in questionnaires.

Questionnaire	High activity choir singers (N = 48)	Low activity choir singers (N = 58)	Control subjects (N = 56)	p value^a^
CFQ total score	24.3 (12.9)	22.0 (11.3)	22.6 (11.8)	0.592
PRMQ total score	32.0 (8.4)	30.3 (7.8)	31.3 (7.6)	0.690
CES-D total score	10.8 (6.0)	9.1 (5.1)	9.5 (5.7)	0.332
SPS total score	84.9 (6.1)	84.3 (6.4)	83.9 (7.4)	0.637
SPS: Attachment scale	14.6 (1.4)	14.6 (1.5)	14.1 (2.1)	0.396
SPS: Social Integration scale	14.5 (1.2)	13.9 (1.5)	13.8 (1.7)	**0.046**
SPS: Reassurance of Worth scale	13.1 (1.7)	13.3 (1.5)	13.4 (1.7)	0.776
SPS: Reliable Alliance scale	14.6 (1.3)	14.6 (1.6)	14.8 (1.4)	0.772
SPS: Guidance scale	14.9 (1.3)	14.8 (1.4)	14.6 (1.7)	0.419
SPS: Opportunity of Nurturance scale	13.2 (1.9)	13.2 (1.6)	13.3 (1.7)	0.779
WHOQOL-Bref: Overall QOL	4.1 (0.6)	4.2 (0.5)	4.3 (0.6)	0.363
WHOQOL-Bref: General Health	3.8 (0.9)	4.2 (0.6)	4.0 (0.8)	**0.029**
WHOQOL-Bref: Physical QOL	16.0 (2.7)	17.0 (2.0)	16.6 (2.5)	0.133
WHOQOL-Bref: Psychological QOL	15.7 (2.0)	16.0 (1.8)	15.8 (2.0)	0.918
WHOQOL-Bref: Social QOL	15.8 (2.5)	16.1 (2.4)	15.6 (2.7)	0.586
WHOQOL-Bref: Environmental QOL	16.6 (2.0)	17.1 (1.7)	17.3 (1.5)	0.135
MusEQ total score	3.2 (0.5)	3.1 (0.4)	2.8 (0.6)	**<0.001**

Data shown as mean (SD).

^a^The p value is the Group effect from univariate ANCOVA with age, female gender and living alone as covariates. Significant effects shown in bold font.

## Discussion

In this study, our aim was to explore whether active participation in choir singing was associated with cognitive, emotional, and social well-being and QOL in healthy older adults. We used a comprehensive set of questionnaires and neuropsychological tests to assess participants’ well-being and cognitive performance in a relatively large sample of older adults. Our hypotheses were that choir singers would have better performance in neuropsychological tests, especially in those measuring executive functions, and that they would experience better emotional, cognitive, and social well-being, compared to controls. These hypotheses were partially supported by the results, which showed that compared to the control group the choir singers had better verbal flexibility and they also experienced better social integration and health and had higher musical engagement in daily life.

In the subcohort study, the main finding was enhanced executive function in the domain of verbal flexibility in the choir singers compared to control subjects. This may reflect the specific verbal-cognitive demands of choir singing, especially the vocal production of lyrics while focusing concurrently on musical structure (the melody and rhythm of the song), auditory perception (perception of own voice and voices of other singers) and action (correcting own voice and adjusting it to others), musical instruction (following conductor’s gestures, anticipating the next words), and emotional expression, which requires flexibility. The result is also in line with the findings of Fu et al. [33] that a 12-week group singing program had a positive effect on phonemic fluency in older adults. Also Mansens et al. [25] reported that music making, which in their study included both singing and playing a music instrument, was associated with better phonemic fluency. Similarly, Hanna-Pladdy and Gajewski [23] found that instrumental musical practice was associated with better phonemic fluency, in addition to better performance also in other tasks measuring cognitive flexibility. Together, these findings suggest that cognitive flexibility seems to be a core function, which is positively affected by musical training and music activity in general during aging. At the neural level, a potential neurobiological mechanism for this effect could be striatal dopamine synthesis, which is known to influence the tuning of networks underlying the preservation of cognitive flexibility in aging [53] and which is increased through the reward and pleasure received from music [15] and during verbal learning [54].

Aside from verbal flexibility, we did not observe benefits of choir singing on any other cognitive domain, either in the neuropsychological tests or in cognitive self-report questionnaires (CFQ, PRMQ). Also in previous studies, findings regarding the effects of choir singing in other cognitive domains have been somewhat mixed. Some studies have reported that choir singing or singing in general is associated with better verbal skills, learning, and memory [25, 33] while others have not. In their randomized controlled trial (RCT), Johnson et al. [38] did not find any cognitive benefits from a community choir intervention for cognitively healthy older adults. Overall, the extent to which choir singing can have positive far transfer effects in the more general domains of executive function, attention, and memory function, still remains unknown.

In the main cohort study, we found that choir singers experienced better general health and social integration compared to the control group. Overall, this is in line with previous studies of older adults reporting that choir singing is associated with reduced loneliness, improved QOL, physical health, and interest in life, and increased general activity [36–39]. Interestingly, in our study, these effects seemed to be mediated by the length of the choir singing activity: better social integration was seen only in the high activity choir singers who had more than 10 years of choir experience whereas better general health was seen only in the low activity choir singers who had started choir singing at older age and had ≤10 years choir experience. As the two choir singer groups were well-matched with regards to how often they currently participated in choir singing as well as in non-musical (cognitive and physical) activities and as the demographic variables in which the groups differed (age, gender, living alone) were controlled for in the analysis, it is yet unclear why the two choir singer groups showed different effects. A potential explanation could be that for the high activity choir singer group, the long-standing choir activity and the personal relationships formed with the other choir members have become an integral part of their social life—a hobby that unites them and keeps them socially connected. In turn, for the low activity choir singers, participation in the choir activity may be more motivated by an aim to maintain better health during aging, as a part of a healthy and active lifestyle.

Our study has some methodological limitations, which should be taken into account when evaluating its findings. First of all, the choir arm of the study is based on a natural sample of older adults participating in choir singing, and therefore we have no experimental control of the choir training, for example in terms of quality and quantity of training, skill level, and goals and motivation, and how these factors could impact the results. On the other hand, this same facet makes the study more representative of real life and provides ecological validity, although the relatively small sample size (especially of the subcohort study) somewhat limits the generalizability of the findings. In recruiting the participants, we made a clear distinction between actual choir singing and other types of group singing (e.g., taking part in singalong sessions, singing as an audience member at church, concerts or sports events etc.), as the goal-oriented and learning-related cognitive component applies more to choir singing. Second, given that this was a cross-sectional study, the results are largely correlational and no conclusions can be made about the causality of findings and the long-term effects of choir singing on neurocognitive aging; for this, more large-scale studies with randomized controlled trial (RCT) and longitudinal cohort designs are needed. The follow-up data from our cohort (study ongoing) may be able to shed more light on this issue. In future studies, also an active control condition (another specific hobby) would be needed. Third, this study focused on cognitive, emotional, and social outcomes and therefore it is not able to determine the potential physiological (e.g., cardiorespiratory), motor (e.g., balance, posture), hormonal (e.g., oxytocin, cortisol), and neural (brain structure and function) effects of choir singing, which would be needed to establish a more comprehensive picture of the role of choir singing in healthy aging.

## Implications

The results of the present cross-sectional study suggest that regular participation in choir singing as a hobby is associated with better verbal flexibility in healthy older adults, but positive effects on other cognitive functions are limited. In addition, long-standing choir participation was linked to better social integration whereas choir activity, which was started at older age, was linked to better general health. Together with previous studies, there is emerging evidence that singing in a choir may provide an accessible and useful way to stave off the negative social-emotional sequelae (e.g., loneliness, social isolation) and cognitive decline, which are typically associated with aging. This is highly important to society because the onset and progress of age-related cognitive decline and mood disorders are known to be closely linked to reduced social interaction [6, 7, 55] and executive dysfunction [2, 3, 8], and there is urgent need for different lifestyle interventions that can be utilized to support healthy aging [5].

## Supporting information

S1 Table Proportion of participants scoring in the impaired range in standardized tests with Finnish normative data. (DOCX)

## References

[1] Salthouse T.A. (2010). Selective review of cognitive aging. *Journal of the International Neuropsychological Society*, 16, 754–760. 10.1017/S1355617710000706 20673381 PMC3637655

[2] Reuter-Lorenz P.A., & Park D.C. (2010). Human neuroscience and the aging mind: A new look at old problems. *Journals of Gerontology*: *Series B*, *Psychological Sciences and Social Sciences*, 65, 405–415. 10.1093/geronb/gbq035 20478901 PMC2883872

[3] Bakkour A., Morris J.C., Wolk D.A., & Dickerson B.C. (2013). The effects of aging and Alzheimer’s disease on cerebral cortical anatomy: Specificity and differential relationships with cognition. *NeuroImage*, 76, 332–344. 10.1016/j.neuroimage.2013.02.059 23507382 PMC4098706

[4] Kubo Y., Hayashi H., Kozawa S., & Okada S. (2018). Relevant factors of depression in dementia modifiable by non-pharmacotherapy: A systematic review. *Psychogeriatrics*, 19, 181–191. 10.1111/psyg.12371 30246316

[5] Kivipelto M., Mangialasche F., & Ngandu T. (2018). Lifestyle interventions to prevent cognitive impairment, dementia and Alzheimer disease. *Nature Reviews Neurology*, 14, 653–666. 10.1038/s41582-018-0070-3 30291317

[6] Boss L., Kang D.H., & Branson S. (2015). Loneliness and cognitive function in the older adult: A systematic review. *International Psychogeriatrics*, 27, 541–553. 10.1017/S1041610214002749 25554219

[7] O’Rourke H.M., Collins L., & Sidani S. (2018). Interventions to address social connectedness and loneliness for older adults: A scoping review. *BMC Geriatrics*, 18, 1–13. 30219034 10.1186/s12877-018-0897-x PMC6139173

[8] Reuter-Lorenz P.A., & Park D.C. (2014). How does it STAC up? Revisiting the Scaffolding Theory of Aging and Cognition. *Neuropsychology Review*, 24, 355–370. 10.1007/s11065-014-9270-9 25143069 PMC4150993

[9] Nguyen L., Murphy K., & Andrews G. (2019). Cognitive and neural plasticity in old age: A systematic review of evidence from executive functions cognitive training. *Ageing Research Reviews*, 53, 100912 10.1016/j.arr.2019.100912 31154013

[10] Liu-Ambrose T., Barha C., & Falck R.S. (2019). Active body, healthy brain: Exercise for healthy cognitive aging. *International Review of Neurobiology*, 147, 95–120. 10.1016/bs.irn.2019.07.004 31607364

[11] Fancourt, D., & Finn, S. (2019). What is the evidence on the role of the arts in improving health and well-being? A scoping review. WHO Health Evidence Network Synthesis Reports. Copenhagen: WHO Regional Office for Europe. 32091683

[12] Noice T., Noice H., & Kramer A.F. (2014). Participatory arts for older adults: A review of benefits and challenges. *Gerontologist*, 54, 741–753. 10.1093/geront/gnt138 24336875 PMC4229893

[13] Särkämö T., & Sihvonen A. J. (2018). Golden oldies and silver brains: Deficits, preservation, learning, and rehabilitation effects of music in ageing-related neurological disorders. *Cortex*, 109,104–123. 10.1016/j.cortex.2018.08.034 30312779

[14] Alluri V., Toiviainen P., Jääskeläinen I. P., Glerean E., Sams M., & Brattico E. (2012). Large-scale brain networks emerge from dynamic processing of musical timbre, key and rhythm. *NeuroImage*, 59, 3677–3689. 10.1016/j.neuroimage.2011.11.019 22116038

[15] Zatorre R.J., & Salimpoor V.N. (2013). From perception to pleasure: Music and its neural substrates. *Proceedings of the National Academy of Sciences of the United States of America*, 110, 10430–10437. 10.1073/pnas.1301228110 23754373 PMC3690607

[16] Benz S., Sellaro R., Hommel B., & Colzato L.S. (2016). Music makes the world go round: The impact of musical training on non-musical cognitive functions—A review. *Frontiers in Psychology*, 6, 2023 10.3389/fpsyg.2015.02023 26779111 PMC4703819

[17] Moreno S., & Bidelman G.M. (2014). Examining neural plasticity and cognitive benefit through the unique lens of musical training. *Hearing Research*, 308, 84–97. 10.1016/j.heares.2013.09.012 24079993

[18] Herholz S.C. & Zatorre R.J. (2012). Musical training as a framework for brain plasticity: Behavior, function, and structure. *Neuron*, 76, 486–502. 10.1016/j.neuron.2012.10.011 23141061

[19] James C.E., Oechslin M.S., Van De Ville D., Hauert C.A., Descloux C., & Lazeyras F. (2014). Musical training intensity yields opposite effects on grey matter density in cognitive versus sensorimotor networks. *Brain Structure and Function*, 219, 353–366. 10.1007/s00429-013-0504-z 23408267

[20] Sluming V., Barrick T., Howard M., Cezayirli E., Mayes A., & Roberts N. (2002). Voxel-based morphometry reveals increased gray matter density in Broca’s area in male symphony orchestra musicians. *NeuroImage*, 17, 1613–1622. 10.1006/nimg.2002.1288 12414299

[21] Rogenmoser L., Kernbach J., Schlaug G., & Gaser C. (2018). Keeping brains young with making music. *Brain Structure & Function*, 223, 297–305. 10.1007/s00429-017-1491-2 28815301

[22] Bugos J.A., Perlstein W.M., McCrae C.S., Brophy T.S., & Bedenbaugh P.H. (2007). Individualized piano instruction enhances executive functioning and working memory in older adults. *Aging & Mental Health*, 11, 464–471. 10.1080/13607860601086504 17612811

[23] Hanna-Pladdy B., & Gajewski B. (2012). Recent and past musical activity predicts cognitive aging variability: Direct comparison with general lifestyle activities. *Frontiers in Human Neuroscience*, 6, 198 10.3389/fnhum.2012.00198 22833722 PMC3400047

[24] Hanna-Pladdy B., & MacKay A. (2011). The relation between instrumental musical activity and cognitive aging. *Neuropsychology*, 25, 378–386. 10.1037/a0021895 21463047 PMC4354683

[25] Mansens D., Deeg D.J., & Comijs H.C. (2018). The association between singing and/or playing a musical instrument and cognitive functions in older adults. *Aging & Mental Health*, 22, 964–971. 10.1080/13607863.2017.1328481 28521542

[26] Zarate J.M. (2013). The neural control of singing. *Frontiers in Human Neuroscience*, 7, 237 10.3389/fnhum.2013.00237 23761746 PMC3669747

[27] Alluri V., Toiviainen P., Lund T.E., Wallentin M., Vuust P., Nandi A.K., et al (2013). From Vivaldi to Beatles and back: Predicting lateralized brain responses to music. *Neuroimage*, 83, 627–636. 10.1016/j.neuroimage.2013.06.064 23810975

[28] Whitehead J., & Armony J. (2018). Singing in the brain: Neural representation of music and voice as revealed by fMRI. *Human Brain Mapping*, 39, 4913–4924. 10.1002/hbm.24333 30120854 PMC6866591

[29] Wang W., Wei L., Chen N., Jones A., Gong G., Liu H. (2019). Decreased grey-matter volume in insular cortex as a correlate of singers’ enhanced sensorimotor control of vocal production. *Frontiers in Neuroscience*, 13, 815 10.3389/fnins.2019.00815 31427924 PMC6688740

[30] European Choral Association (2015). Singing Europe Report 2015. Retrieved on April 3rd 2020 from: https://europeanchoralassociation.org/wp-content/uploads/2019/01/singingeurope_report.pdf.

[31] Williams E., Dingle G.A., & Clift S. (2018). A systematic review of mental health and wellbeing outcomes of group singing for adults with a mental health condition. *European Journal of Public Health*, 28, 1035–1042. 10.1093/eurpub/cky115 29982515

[32] Bernardi N.F., Snow S., Peretz I., Orozco Perez H.D., Sabet-Kassouf N., Lehmann A. (2017). Cardiorespiratory optimization during improvised singing and toning. *Scientific Reports*, 7, 8113 10.1038/s41598-017-07171-2 28808334 PMC5556092

[33] Fu M.C., Belza B., Nguyen H., Logsdon R., & Demorest S. (2018). Impact of group-singing on older adult health in senior living communities: A pilot study. *Archives of Gerontology and Geriatrics*, 76, 138–146. 10.1016/j.archger.2018.02.012 29518671

[34] Kreutz G., Bongard S., Rohrmann S., Hodapp V., & Grebe D. (2004). Effects of choir singing or listening on secretory immunoglobulin A, cortisol, and emotional state. *Journal of Behavioral Medicine*, 27, 623–635. 10.1007/s10865-004-0006-9 15669447

[35] Stone N.L., Millar S.A., Herrod P.J., Barrett D.A., Ortori C.A., Mellon V.A., et al (2018). An analysis of endocannabinoid concentrations and mood following singing and exercise in healthy volunteers. *Frontiers in Behavioral Neuroscience*, 12, 269 10.3389/fnbeh.2018.00269 30534062 PMC6275239

[36] Cohen G.D., Perlstein S., Chapline J., Kelly J., Firth K.M., & Simmens S. (2006). The impact of professionally conducted cultural programs on the physical health, mental health, and social functioning of older adults. *Gerontologist*, 46, 726–734. 10.1093/geront/46.6.726 17169928

[37] Coulton S., Clift S., Skingley A., & Rodriguez J. (2015). Effectiveness and cost-effectiveness of community singing on mental health-related quality of life of older people: Randomised controlled trial. *British Journal of Psychiatry*, 207, 250–255. 10.1192/bjp.bp.113.129908 26089304

[38] Johnson J.K., Stewart A.L., Acree M., Nápoles A.M., Flatt J.D., Max W.B., et al (2020). A community choir intervention to promote well-being among diverse older adults: Results from the Community of Voices trial. *Journals of Gerontology*: *Series B*, *Psychological Sciences and Social Sciences*, 75, 549–559. 10.1093/geronb/gby132 30412233 PMC7328053

[39] Johnson J.K., Louhivuori J., Stewart A.L., Tolvanen A., Ross L., & Era P. (2013). Quality of life (QOL) of older adult community choral singers in Finland. *International Psychogeriatrics*, 25, 1055–1064. 10.1017/S1041610213000422 23574947 PMC3748797

[40] Nasreddine Z.S., Phillips N.A., Bédirian V., Charbonneau S., Whitehead V., Collin I., et al (2005). The Montreal Cognitive Assessment, MoCA: A brief screening tool for mild cognitive impairment. *Journal of American Geriatric Society*, 53, 695–699. 10.1111/j.1532-5415.2005.53221.x 15817019

[41] Lezak M.D., Howiesson D.B., Bigler E.D., & Tranel D. (2012). *Neuropsychological Assessment*. 5th ed New York, NY: Oxford University Press.

[42] Martin N., Kohen F., Kalinyak-Fliszar M., Soveri A., & Laine M. (2012). Effects of working memory load on processing of sounds and meanings of words in aphasia. *Aphasiology*, 26, 462–493. 10.1080/02687038.2011.619516 22544993 PMC3335394

[43] Wechsler D. (2008). *Wechsler Adult Intelligence Scale*, 4th ed Pearson Assessment (Finnish version: Psykologien Kustannus oy, Helsinki, 2012).

[44] Kessels R., Zandvoort M., Postma A., Kappelle L., Haan E. (2000). The Corsi Block-Tapping Task: Standardization and Normative Data. *Applied Neuropsychology*, 7:4, 252–258. 10.1207/S15324826AN0704_8 11296689

[45] Wechsler D. (1997). *Wechsler Memory Scale*, 3rd ed San Antonio: The Psychological Corporation (Finnish version: Psykologien Kustannus oy, Helsinki, 2008).

[46] Broadbent D.E., Cooper P.F., FitzGerald P., & Parkes K.R. (1982). The Cognitive Failures Questionnaire (CFQ) and its correlates. *British Journal of Clinical Psychology*, 21, 1–16. 10.1111/j.2044-8260.1982.tb01421.x 7126941

[47] Smith G., Della Sala S., Logie R.H., & Maylor E.A. (2000). Prospective and retrospective memory in normal ageing and dementia: A questionnaire study. *Memory*, 8, 311–321. 10.1080/09658210050117735 11045239

[48] Radloff L.S. (1977) The CES-D scale: A self-report depression scale for research in the general population. *Applied Psychological Measurement*, 1, 385–401.

[49] Cutrona C.E., & Russell D.W. (1987). The provisions of social relationships and adaptation to stress. *Advances in Personal Relationships*, 1, 37–67.

[50] Group WHOQOL (1998). Development of the World Health Organization WHOQOL-BREF quality of life assessment. *Psychological Medicine*, 28, 551–558. 10.1017/s0033291798006667 9626712

[51] Vanstone A.D., Wolf M., Poon T., & Cuddy L.L. (2016). Measuring engagement with music: Development of an informant-report questionnaire. *Aging & Mental Health*, 20, 474–484. 10.1080/13607863.2015.1021750 25811870

[52] Valenzuela M.J., & Sachdev P. (2007). Assessment of complex mental activity across the lifespan: Development of the Lifetime of Experiences Questionnaire (LEQ). *Psychological Medicine*, 37, 1015 10.1017/S003329170600938X 17112402

[53] Berry A.S., Shah V.D., Baker S.L., Vogel J.W., O’Neil J.P., Janabi M., et al (2016). Aging affects dopaminergic neural mechanisms of cognitive flexibility. *Journal of Neuroscience*, 36, 12559–12569. 10.1523/JNEUROSCI.0626-16.2016 27807030 PMC5157103

[54] Ripollés P., Ferreri L., Mas-Herrero E., Alicart H., Gómez-Andrés A., Marco-Pallares J., et al (2018). Intrinsically regulated learning is modulated by synaptic dopamine signaling. *Elife*, 7, 10.7554/eLife.38113 30160651 PMC6133552

[55] Valtorta N., Kanaan M., Gilbody S., Hanratty B. (2018). Loneliness, social isolation and risk of cardiovascular disease in the English Longitudinal Study of Ageing. *European Journal of Preventive Cardiology*, 25, 1387–1396. 10.1177/2047487318792696 30068233

