# Supplementary material for: Beneficial effects of choir singing on cognition and well-being of older adults: Evidence from a cross-sectional study
Source: PLoS One. 2021 Feb 3;16(2):e0245666. doi: 10.1371/journal.pone.0245666 (PMC7857631; doi:10.1371/journal.pone.0245666)
Supplement: S1 Table — (DOCX) [file pone.0245666.s001.docx]

| **Domain** | **Measure** | **% of subjects in impaired range** | **Difference between groups (X^2^)** |
| --- | --- | --- | --- |
|  |  | Choir (N=39) Control (N=35) |  |
| Processing speed | WAIS-IV Visual search | 17.9 17.1 | 0.93 |
|  | WAIS-IV Coding | 10.3 0 | 0.05 |
| Working memory | WAIS-IV Digit span | 5.1 8.6 | 0.56 |
| Problem solving | WAIS-IV Arithmetic | 23.1 22.9 | 0.92 |
| EM:  Immediate | WMS-III Word lists | 20.5 11.4 | 0.34 |
| EM:  Delayed | WMS-III Word lists | 17.9 17.1 | 0.93 |
| Verbal skills | WAIS-IV Vocabulary | 7.7 14.3 | 0.36 |

**S1 Table.** Proportion of participants scoring in the impaired range in standardized tests with Finnish normative data. Abbreviations: EF=executive function, EM=episodic memory.
