# Supplementary material for: Beneficial effects of choir singing on cognition and well-being of older adults: Evidence from a cross-sectional study
Source: PLoS One. 2021 Feb 3;16(2):e0245666. doi: 10.1371/journal.pone.0245666 (PMC7857631; doi:10.1371/journal.pone.0245666)
Supplement: Attachment — Submitted filename: Response to Reviewers_PONE-D-20-19456.docx [file pone.0245666.s002.docx]

Pentikäinen et al. Beneficial effects of choir singing on cognition and well-being of older adults: evidence from a cross-sectional study (PONE-D-20-19456)

**Response to Reviewers**

We would like to thank the Reviewers for their valuable comments, which have greatly helped us to improve the manuscript. Below, we have listed our responses to each point made by the Reviewers.

**Reviewer 1**

1. Please consider including a reference to the study design in the title, i.e. cross-sectional study.

**Response:** We agree that adding the study type to the title is a good idea and can avoid potential misunderstanding. We have now changed the title to “Beneficial effects of choir singing on cognition and well-being of older adults: a cross-sectional study”

2. By “long-time” and “short-time” choir experience do you mean “long-term” and “short-term”?

**Response:** Thank you for noting this. In hindsight, we agree that the terms “long-time” and “short-time” were somewhat awkward and potentially misleading. The suggested terms “long-term” and “short-term” are better, but in our view, “short-term” refers to a time period shorter than the one we mean here (choir singing experience ranging from 1 to 10 years); also the Merriam-Webster dictionary defines “short-term” as something “occurring over or involving a relatively short period of time”, often less than 6 months / 1 year. In revising the terms, we opted to use the terminology used previously by Hanna-Pladdy and MacKay (2011, *Neuropsychology*) who divided their older adult musicians to low activity (1–9 years) and high activity (>10 years) groups based on years of musical experience throughout their life span. Thus, we have now changed “long-term” and “short-term” to “low activity” and “high activity”, respectively, across the manuscript.

3. Introduction is clear and succinct and supported by appropriate current references. Suggest writing “executive function” out in full rather than abbreviate to EF. A clear summary of current published knowledge in the area and justification for the study.

**Response:** Thank you for the positive feedback. We have now replaced the EF abbreviation with “executive function” across the manuscript.

4. Discussion and Implications are comprehensive and appropriate reflect on findings and study limitations in relation to previous research. Possible typo on p12 line 359 “positive effects on other cognitive functions…”

**Response:** There was indeed a typo, which we have now corrected.

**Reviewer 2**

1. The dichotomy between long-time (>10 years, N = 48) and short time (≤10 years, N = 58) choir singers seems arbitrary and is not well explained. In fact, the differences in the results are not easily explainable and remain speculative. I miss a minimum criterium of choir singing (e.g., include only participants who have at least two or three years of choir singing). In order to find beneficial effects, choir singing has to extend over a certain time span. Then I would enter the duration of choir singing as a continuous variable instead as a categorical and see the effects.

**Response:** Our decision to use dichotomous classification was motivated by (i) previous studies utilizing the same approach in musicians and their findings that the duration of musical activity has an impact on the cognitive benefits incurred by music, (ii) the distribution of years of choir singing in our sample, and (iii) practical considerations of equal group sizes for statistical analysis.

i. Previously, in their study of healthy older adults, Hanna-Pladdy and MacKay (2011, *Neuropsychology*) divided the musician sample to low (1–9 years) and high (>10 years) activity group based on years of musical experience throughout the life span. They showed that compared to non-musician controls, only the high activity musicians had better performance in cognitive tests. Also other studies have shown that musical experience acquired earlier in life vs. more recently can have differential effects on cognitive functioning (Hanna-Pladdy & Gajeweski, 2012; Strong & Mast, 2018). In light of the comment of Reviewer 1, we have now adopted the terms used by Hanna-Pladdy and MacKay (2011) and replaced “short-time” with “low activity” and “long-time” with “high activity”, and cite this study also in the Methods section.
ii. In our sample, years of choir singing did not follow a normal distribution (see figure below), which is problematic for its inclusion as a parametric (continuous) variable in the analyses. The median value was 10 years, which was chosen as a natural cut-off point for the group classification. In the low activity choir singers, the mean of years of singing was 6.3 years and SD 2.8 years. In the high activity singers, these were 27.3 years and 14.2 years, respectively. We have added this information in the manuscript (see Table 2).
iii. Given that in the main cohort, the sample sizes of the controls (N=56) and choir singers (N=106) were unbalanced, which leads to unequal variances between samples that violates the assumption of equal variances in ANOVA and affects statistical power and Type I error rates (e.g., Rusticus & Lovato, 2014, *Practical Assessment, Research & Evaluation*). Including years of choir singing as a classifying variable results in relatively balanced group sizes (controls: N=56, low activity singers: N=58, high activity singers: N=48), differing only by 4%–11% from the optimal group size (162/3=54), as opposed to the 31% difference from optimal if two groups were used (N=56 and N=106; 162/2=81). Thus, our approach avoids the unequal sample size problem.

Regarding the minimum amount of choir singing, we actually included also a “minimum 1 year” criterion when recruiting the choir singers. Thus, all the choir singers had to be (1) currently singing in a choir and (2) having sung in a choir for at least one year. Unfortunately, this important information was omitted from the original manuscript by mistake; it has now been added to the Methods section.

2. I would not enter arithmetic as a working memory test. Though arithmetic relies on working memory, it requires also other cognitive domains (e.g., procedural knowledge, conceptual knowledge). Please list arithmetic as a separate domain.

**Response:** We agree that including arithmetic in the working memory domain is somewhat misleading (it was originally included there as it is belongs to the Working memory index of WAIS-IV, but since we are also using the visual span here, which is not from WAIS-IV, there is no need to adhere to the WAIS-IV guideline). We have now included the WAIS-IV Arithmetic score as a separate domain of Problem solving and reanalyzed the data for the Working memory (now WAIS-IV Digit span and FAT Visual span subtest) and Problem solving domains (WAIS-IV Arithmetic). This does not change the results (neither domain differs between the choir singers and controls).

3. Table 3: Are these values in the average of standardized norms? Above/below? Are there differences between groups (proportion of participants scoring in the impaired range)?

**Response:** Originally, we chose to report the raw scores (or their sums for the cognitive domains) of the neuropsychological tests because not all tests were standardized and have adequate Finnish normative data. For those tests which are standardized and which have adequate normative data, and for which a cut-off value for impaired performance can be established (visual search, coding, digit span, arithmetic, word lists, vocabulary), there were no differences between the choir singer and control groups on the proportion of participants scoring in the impaired range (see table below). Even though a participant may have scored in the impaired range in one or more of the tests, they did not have any diagnosed neurodegenerative diseases, which was our inclusion criterion.

| **Domain** | **Measure** | **% of subjects in impaired range** | **Difference between groups (X^2^)** |
| --- | --- | --- | --- |
|  |  | Choir (N=39) Control (N=35) |  |
| Processing speed | WAIS-IV Visual search | 17.9 17.1 | 0.93 |
|  | WAIS-IV Coding | 10.3 0 | 0.05 |
| Working memory | WAIS-IV Digit span | 5.1 8.6 | 0.56 |
| Problem solving | WAIS-IV Arithmetic | 23.1 22.9 | 0.92 |
| EM:  Immediate | WMS-III Word lists | 20.5 11.4 | 0.34 |
| EM:  Delayed | WMS-III Word lists | 17.9 17.1 | 0.93 |
| Verbal skills | WAIS-IV Vocabulary | 7.7 14.3 | 0.36 |

Abbreviations: EF=executive function, EM=episodic memory
